# A novel association of pyroptosis-related gene signature with the prognosis of hepatocellular carcinoma

**DOI:** 10.3389/fonc.2022.986827

**Published:** 2022-10-04

**Authors:** Yuyao Li, Yue Li, Xuemei Zhang, Xiangjuan Duan, Hai Feng, Zhuo Yu, Yueqiu Gao

**Affiliations:** ^1^ Institute of Infectious Disease, Shuguang Hospital Affiliated to Shanghai University of Traditional Chinese Medicine, Shanghai, China; ^2^ Department of Liver Disease, Shuguang Hospital Affiliated to Shanghai University of Traditional Chinese Medicine, Shanghai, China

**Keywords:** pyroptosis, hepatocellular carcinoma, prognosis, risk model, immune infiltration

## Abstract

**Background:**

Hepatocellular carcinoma (HCC) is one of the global leading lethal tumors. Pyroptosis has recently been defined as an inflammatory programmed cell death, which is closely linked to cancer progression. However, the significance of pyroptosis-related genes (PRGs) in the prognosis of HCC remains elusive.

**Methods:**

RNA sequencing (RNA-seq) data of HCC cases and their corresponding clinical information were collected from the Cancer Genome Atlas (TCGA) database, and differential PRGs were explored. The prognostic PRGs were analyzed with univariate COX regression and the least absolute shrinkage and selection operator (LASSO) Cox regression analysis to build a prognostic model in the TCGA training cohort. The predictive model was further validated in the TCGA test cohort and ICGC validation cohort. Differential gene function and associated pathway analysis were performed by Gene ontology (GO) and Kyoto Encyclopedia of Gene and Genomes (KEGG). Single-sample gene set enrichment analysis (ssGSEA) was used to identify distinct immune cell infiltration. The mRNA and protein expression of prognostic PRGs was examined by quantitative RT-qPCR and immunohistochemistry.

**Results:**

We identified 46 PRGs that were differentially expressed between normal and HCC tissues in a TCGA cohort, and HCC patients could be well categorized into two clusters associated with distinct survival rates based on expression levels of the PRGs. A three-PRG prognostic model comprising *CHMP4A, HMGB1 and PLK1* was constructed in the training cohort, and HCC patients could be classified into the high- and low-risk subgroups based on the median risk score. High-risk patients exhibited shorter overall survival (OS) than low-risk ones, which was validated in the test cohort and ICGC validation cohort. The risk score of this model was confirmed as an independent prognostic factor to predict OS of HCC patients. GO, KEGG and ssGSEA demonstrated the differential immune cell infiltrations were associated with the risk scores. The higher expression of *CHMP4A, HMGB1 and PLK1* were validated in HCC compared to normal *in vivo* and *in vitro*.

**Conclusion:**

The three-PRG signature (CHMP4A, HMGB1, and PLK1) could act as an independent factor to predict the prognosis of HCC patients, which would shed light upon a potent therapeutic strategy for HCC treatment.

## Introduction

Hepatocellular carcinoma (HCC) is the fourth leading cause of cancer-related death (1). In Asian, the 5-year survival rate of HCC patients has recently been reported to drop to 12% (2). Hepatitis B virus, hepatitis C virus, non-alcoholic steatohepatitis (NASH), diabetes mellitus, excessive alcoholic consumption, cirrhosis, genetic predisposition and aflatoxin contamination of food crops are the etiology of HCC (3). For lacking the early effective diagnostic methods, HCC is mostly detected in the advanced stage with poor therapeutic outcome (4). Despite the progress achieved in HCC therapy, the fact that most patients suffer various degrees of relapse after treatment is still a big medical problem (5). The limitation on HCC therapy urges to establish the novel reliable models to evaluate the prognosis for optimization of therapeutic strategy.

Programmed cell death (PCD) is a pathological form of cell death that is evoked by abnormal stimuli, and includes necrosis, apoptosis and pyroptosis (6). Pyroptosis, also known as cellular inflammatory necrosis, is a new form of PCD characterized with the activation of strong inflammatory response *via* the production of danger-associated signaling molecules and cytokines, which is closely linked to cancer progression (7). Compared with apoptosis that shows immunological inertness in the cell death, pyroptosis is characterized with the induction of immune inflammatory cell death by releasing the inflammatory factors (8). The formation of inflammatory body, or inflammasome, is the key event in pyroptosis, which is mediated by gasdermin family that is consisted of various pore-forming proteins (9, 10). Gasdermin D (GSDMD) and gasdermin E (GSDME) are the important members that play the role in the induction of pyroptosis by triggering amplified inflammatory response. In this process, distinct pyroptotic caspase molecules provoke the activities of GSDMD and GSDME through the involvement of two different enzyme-regulated pathways. Canonical pathway is mediated by caspase-1 activation, while non-canonical pathway is dependent on caspase-4/5/11 activation (11, 12). In detail, active caspase-1 targets GSDMD and GSDME as the substrates and cleaves them into N-terminal domain and C-terminal domain for cell membrane perforation. In comparison, caspase-4/5/11 is activated by binding to lipopolysaccharide (LPS) and specially cleaves GSDMD to mediate the dissolution of cell membrane. Meanwhile, activated caspase-4/5/11 also stimulates the release of ATP and the assembly of channel P2X7 for the pore formation on cell membrane (10, 13). After being cleaved, GSDMD and GSDME form pores in the cell membrane, and cell contents are released through the membrane pores, leading to the inflammatory cell injury and death.

A close relationship exists between pyroptosis and various human diseases, especially malignant tumors (14). However, pyroptosis may show the paradoxical effect on the pathogenesis of tumors (15). On one hand, pyroptosis *per se* and pyroptosis-induced signaling molecules and cytokines inhibit the proliferation and metastasis of tumor cells. Some studies have suggested that pyroptosis evoked robust antitumor immunity through the induction of inflammation, and synergized with immune checkpoint blockade (16, 17). GSDME, capable of converting non-inflammatory apoptosis to pyroptosis, has been reported to trigger antitumor immunity, while reduced GSDME is associated with decreased breast cancer survival (18). On the other hand, the accumulation of inflammasome produced in pyroptosis is conducive to the building of the suitable microenvironment for tumor growth (15). Evidence has indicated that the long-term exposure of tissues and cells to the inflammatory environment increases the risk of cancer. The activation of pyroptosis leads to the generation of inflammatory mediators IL-1 and IL-18, which could promote the occurrence of cancer in many ways (19). In HCC, NLRP3 inflammasome has been demonstrated to induce pyroptosis *via* the activation of GSDMD upon caspase 1-mediated cleavage, which promote cell death and exert antitumor effect on the cancer cells (20). These studies highlight the importance of pyroptosis in the development of cancer, and the expression profile of pyroptosis-related molecules can reflect the pathological status of cancer.

Recent studies demonstrated that the application of pyroptosis-related genes (PRGs) signature has shown the benefit for the prediction of prognosis and the improvement of therapy in ovarian cancer (21), gastric cancer (22) and lung adenocarcinoma (23). However, the prognostic value of pyroptosis in HCC has not yet been elucidated. Thus, we conducted a systematic analysis to investigate the expression level of PRGs between normal and HCC tissues, explore the role of these genes in the evaluation of survival rate of patients, and examine the correlation between pyroptosis and immune response to tumor.

## Materials and methods

### Datasets

RNA sequencing (RNA-seq) data of 418 HCC cases and their corresponding clinical information were obtained from the Cancer Genome Atlas (TCGA) database (24) (https://portal.gdc.cancer.gov/repository). Gene expressions of the 58 normal tissues and 407 tumor tissues in these patients were sorted and standardized using the “limma” package in R software. The clinical parameters included overall survival (OS), age, gender, tumor grade, clinical stage, and pathological grade of patients. After excluding patients with missing survival information, a total of 404 qualified cases were kept and randomly divided into the training and test cohorts. Besides, transcriptomics data of 231 HCC patients (LIRI-JP) and their corresponding clinical features were obtained from the ICGA database (https://dcc.icgc.org/projects/LIRI-JP).

### Identification of differentially expressed PRGs

55 PRGs acquired from the previous studies are listed in **Table 1** (13, 19, 25, 26), and were screened for the differently expressed genes (DEGs) by comparing normal with tumor samples using the “limma” package in R software. Significant difference for gene expression was defined as false discovery rate (FDR) <0.05 and |log_2_FC|>1, and visualized in the heatmap plotted with the “pheatmap” package. A protein-protein interaction (PPI) network for the DEGs was determined with Search Tool for the Retrieval of Interacting Genes (STRING version11.0, https://string-db.org/). A co-expression relationship between the DEGs was analyzed using the “reshape2” and “igraph” packages in R software.

**Table 1:** Pyroptosis-related Gene Signature with the Prognosis of Hepato cellular Carcinoma.

Gene	Description
BAK1	BCL2 Antagonist/Killer 1
BAX	BCL2 Associated X
CASP1	Caspase 1
CASP3	Caspase 3
CASP4	Caspase 4
CASP5	Caspase 5
CHMP2A	Charged Multivesicular Body Protein 2A
CHMP2B	Charged Multivesicular Body Protein 2B
CHMP3	Charged Multivesicular Body Protein 3
CHMP4A	Charged Multivesicular Body Protein 4A
CHMP4B	Charged Multivesicular Body Protein 4B
CHMP4C	Charged Multivesicular Body Protein 4C
CHMP6	Charged Multivesicular Body Protein 6
CHMP7	Charged Multivesicular Body Protein 7
CYCS	Cytochrome C
ELANE	Elastase, Neutrophil Expressed
GSDMD	Gasdermin D
GSDME	Gasdermin E
GZMB	Granzyme B
HMGB1	High Mobility Group Box 1
IL18	Interleukin 18
IL1A	Interleukin 1 Alpha
IL1B	Interleukin 1 Beta
IRF1	Interferon Regulatory Factor 1
IRF2	Interferon Regulatory Factor 2
TP53	Tumor Protein P53
TP63	Tumor Protein P63
AIM2	Absent In Melanoma 2
CASP6	Caspase 6
CASP8	Caspase 8
CASP9	Caspase 9
GPX4	Glutathione Peroxidase 4
GSDMA	Gasdermin A
GSDMB	Gasdermin B
GSDMC	Gasdermin C
IL6	Interleukin 6
NLRC4	NLR Family CARD Domain Containing 4
NLRP1	NLR Family CARD Domain Containing 1
NLRP2	NLR Family CARD Domain Containing 2
NLRP3	NLR Family CARD Domain Containing 3
NLRP6	NLR Family CARD Domain Containing 6
NLRP7	NLR Family CARD Domain Containing 7
NOD1	Nucleotide Binding Oligomerization Domain Containing 1
NOD2	Nucleotide Binding Oligomerization Domain Containing 2
PJVK	Pejvakin
PLCG1	Phospholipase C Gamma 1
PRKACA	Protein Kinase CAMP-Activated Catalytic Subunit Alpha
PYCARD	PYD And CARD Domain Containing
SCAF11	SR-Related CTD Associated Factor 11
TIRAP	TIR Domain Containing Adaptor Protein
TNF	Tumor Necrosis Factor
GZMA	Granzyme A
PLK1	Polo Like Kinase 1
DHX9	DExH-Box Helicase 9
DFNA5	Deafness autosomal dominant 5

### Consensus clustering

The clustering analysis of pyroptosis-related DEGs expression patterns to explore the connection between DEGs expression and survival status was conducted *via* the “ConsensuClusterPlus” package. The matrix heatmap showing the correlation of DEGs expression with different clusters was plotted with the “pheatmap” package. Kaplan-Meier analysis was conducted *via* the “survminer” package to evaluate OS difference between the clusters.

### Establishment and validation of DEGs prognostic model

The expression level of DEGs was standardized in the training cohort, and the correlation between each DEG and survival status was assessed by univariate Cox regression analysis for screening the candidates with significant prognostic value. The candidates were further identified in the construction of prognostic model *via* LASSO Cox regression analysis using the “glmnet” R package. The qualified candidates and their regression coefficient were retained, and the penalty parameter (λ) was determined by the minimum criteria. The risk score of DEG prognostic signature in each sample was calculated with the formula of Σ*X_i_Y_i_*, which *X* represents the expression level of each gene, and *Y* represents the corresponding regression coefficient. HCC patients were divided into high- and low-risk groups based on the median risk score.

The principal component analysis (PCA) and t-distributed stochastic neighbor embedding (t-SNE) were separately conducted to study the distribution of two risk groups in terms of gene expression in the prognostic model. The OS between two groups was evaluated by Kaplan-Meier analysis using the “survival” package, and was plotted with the “survminer” package. The receiver operating curve (ROC) analysis was conducted using “time ROC” package, and the area under curve (AUC) was calculated at 1, 2, 3 year to evaluate the accuracy of the prognostic model. Furthermore, the test cohort and ICGC external validation cohort were utilized for the validation of the prognostic model. Risk score of each sample was calculated according to the established formula. The patients were sorted into high- and low-risk groups based on the median risk score in the training cohort, and OS between two groups was assessed by Kaplan-Meier analysis. The expression levels of the prognostic DEGs were validated using GEPIA (http://gepia.cancer-pku.cn), which is a new resource for gene expression analysis based on tumor and normal samples from the TCGA and Genotype-Tissue Expression (GTEx) databases (27). Protein expression of the DEGs in HCC tumor and non-tumor tissues was evaluated by the human protein atlas (https://www.proteinatlas.org/) (28).

### Independent prognostic analysis of the risk score

Clinical information of HCC patients was extracted from the training cohort and the test cohort. Univariate and multivariate Cox regression analyses were employed to evaluate the clinical variables in combination with the risk score in DEGs prognostic model, and forest maps were plotted to indicate the independent prognostic value of the risk score.

### Functional enrichment analysis

Based on the median risk score, HCC patients in the training cohort were divided into high- and low-risk groups, and the DEGs between the two subgroups were taken for the functional enrichment analysis. The DEGs were screened by virtue of |log_2_FC|>2 and FDR<0.05. Gene ontology (GO) and Kyoto Encyclopedia of Gene and Genomes (KEGG) analyses were performed using the “clusterProfiler” package of R software. Ultimately, the single-sample gene set enrichment analysis (ssGSEA) was performed with “gsva” package to evaluate the content of infiltrating immune cells and the activity of immune-related pathways between the low- and high-risk groups.

### Cell culture

Human Huh7 HCC cells and HL-7702 normal hepatocytes, and mouse Hepa1-6 HCC cells were cultured in DMEM high-glucose medium (Gibco) containing 10% fetal bovine serum (FBS; Hyclone). The cells were cultured at 37 °C in 5% CO2-containing humidified incubator.

### Establishment of HCC mouse model

4-week-old male C57BL/6 mice were ordered from SLAC Laboratory Animal Co. Ltd (Shanghai, China) and received appropriate care. All animals were housed in specific pathogen free conditions on a 12 h light/dark cycle with free access to water and food. The mice were randomized into control group and test one (n=5 per group). In orthotopic HCC model, mice were anesthetized and the livers were exposed. The 5×10^6^ of Hepa1-6 cells were slowly injected into liver strictly following the aseptic principle during the operation. After 2 weeks, the mice were euthanized, and the tumors were harvested for further experiments.

### Reverse transcription and quantitative polymerase chain reaction

Total RNAs isolated from cells with Trizol reagent (invitrogen) were quantified, and reversely transcribed into cDNA using cDNA Reversed Transcription kit (Invitrogen). Equal amount of cDNA was amplified for the detection of *CHMP4A*, *HMGB1* and *PLK1* levels using the SYBR Green qPCR Master Mix (Fermentas) according to the manufacturer’s instructions. Each sample was performed in triplicate and the transcription level was represented as mean ± SEM. GAPDH was used as a house-keeping control gene. The primers are listed in **Table 2**.

**Table 2:** Primer name and primer sequence.

Primer Name	Primer sequence (5' to 3')
m HMGB1-qF	CAAGAAGTGCTCAGAGAGGTGGAAG
m HMGB1-qR	GGGCGGTACTCAGAACAGAACAAG
m PLK1-qF	GTGCCACCTTAGTGACTTGCTACAG
m PLK1-qR	AGTGAGATAGGACTCCGTGCCATC
m CHMP4A-qF	TCCTTCGGCCTTCTTCCTCTTCTG
m CHMP4A-qR	CCTCCTCCTCCTCATCCTCTTCATC
h HMGB1-qF	TCCTTCGGCCTTCTTCCTCTTCTG
h HMGB1-qR	CCTCCTCCTCCTCATCCTCTTCATC
h PLK1-qF	GTGCCTAAGTCTCTGCTGCTCAAG
h PLK1-qR	TCAGGCTCAGTCAGGGCTTTCC
h CHMP4A-qF	TGGCACAAACTGACGGGACATTATC
h CHMP4A-qR	CCAGCAGTTCATCCTCATCCACATC

### Immunohistochemistry

Archived tumor tissues and adjacent non-tumor tissues from HCC patients were used for immunohistochemistry assay as described. Briefly, the sliced sections of formalin-fixed paraffin-embedded tissues were deparaffinized, rehydrated and rinsed in distilled water. The endogenous peroxidase activity was blocked by immersing the slides in 3% hydrogen peroxide. Tissues sections were incubated with primary antibodies at 4°C overnight, followed by incubation with anti-rabbit or anti-mouse secondary antibodies at 37°C for 30 min. The primary antibodies used for IHC staining were rabbit anti-human CHMP4A (Cat# PA5-117867, Invitrogen, 1:100), mouse anti- human HMGB1 (Cat#ab77302, Abcam, 1:200) and mouse anti-human PLK1 (Cat#37-7000, Invitrogen, 1:100).

### Statistical analysis

Data analysis and graphics in the training and test cohort were executed by R software (4.1.0). One-way analysis of variance test was applied to compare the gene expression levels between normal and HCC tissues. The Pearson chi-square test was used to compare the categorical variables in clinical information. Kaplan-Meier analysis with the log-rank Mantel-Cox test was performed to determine the OS of patients between the high- and low-risk subgroups. Univariate and multivariate Cox regression were applied to assess the independent prognostic value of the risk score. The Mann-Whitney test was performed to evaluate the difference of immune cell infiltration and immune pathway activity between the high- and low-risk subgroups. Student *t*-test was performed by GraphPad Prism 8 (GraphPad software) to examine the difference of gene expression between HCC and normal groups *in vitro* and *in vivo* experiments. The data were represented as mean ± SEM in triplicate.

## Results

### Identification of DEGs between normal and tumor tissues

**Figure 1:**
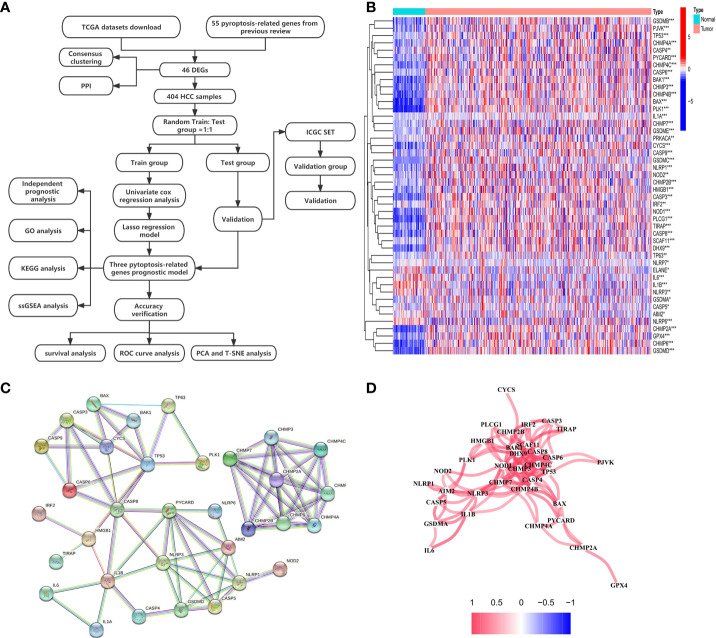
Investigation of expression levels of 46 DEGs and their mutual interactions. **(A)** A workflow diagram exhibits the whole analytic procedures. **(B)** A heatmap shows the expression levels of DEGs between normal and tumor tissues. *P < 0.05; **P < 0.01; ***P < 0.001. **(C)** A PPI network was constructed to display the interactions of the DEGs. **(D)** The correlation network of the DEGs was built. Red line represented positive correlation. The depth of the colors reflected the strength of the relevance.

The expression levels of 55 PRGs were assessed in a TCGA cohort, and 46 DEGs were identified between 58 normal and 407 tumor tissues (**Figure 1A**) (*P*<0.05). Most genes were highly enriched in tumor tissues, while only 4 genes including *ELANE*, *IL6*, *IL1B* and *NLRP3* were upregulated in normal tissues. The transcription levels of these DEGs between two groups were shown in a heatmap (**Figure 1B**). Moreover, a PPI analysis was performed to investigate the interaction of these DEGs. The minimum required interaction score was set at 0.9 as the highest confidence, and we determined that *CHMP7*, *CHMP3*, *CHMP4C*, *CASP8*, *SCAF11*, *CASP4*, *TP53* and *NOD1* serve as the central genes in the PPI network (**Figure 1C**). In addition, the correlation network analysis for all DEGs exhibited obvious positive correlation between either two of any of DEGs in the network (**Figure 1D**). Taken together, we identified 46 PRGs that were differentially expressed between normal and HCC tissues in a TCGA cohort.

### Tumor classification based on the DEGs

**Figure 2:**
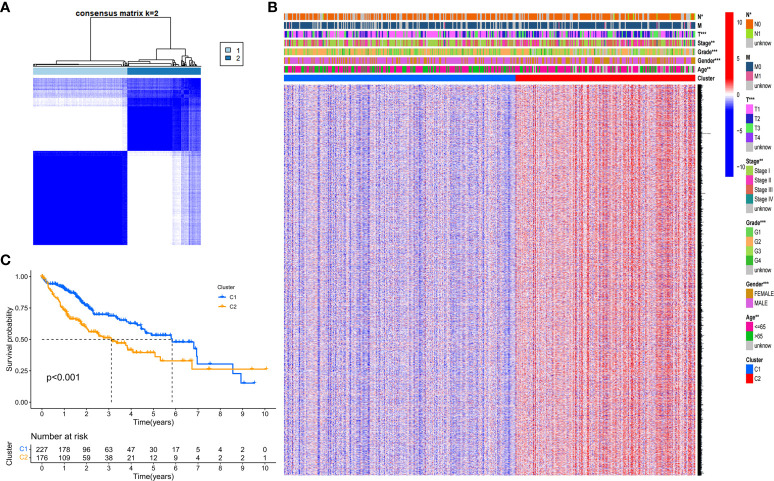
Tumor classification based on the DEGs. **(A)** HCC patients were stratified into two clusters in the consensus clustering matrix (k = 2). **(B)** A heatmap shows gene expression profile and the clinical characters in the two clusters. **(C)** Kaplan–Meier survival curves show the OSs of the two clusters. *P<0.05; **P<0.01; ***P<0.001.

We next explored the correlation between the expression of the 46 DEGs and HCC subtypes by conducting consensus clustering analysis for the 407 HCC patients in the TCGA dataset. These HCC patients could be preferentially divided into two clusters based on the 46 DEGs, displaying the highest intragroup correlation and the low intergroup correlation (**Figure 2A**). The gene expression profile and clinical characters such as the phase of tumor metastasis (N0-N1, M0-M1, T1-T4), clinical stage (stage I- stage IV), degree of tumor differentiation (G1-G4), gender (female or male) and age (≤65 or >65 years), were presented in a heatmap in term of the clustering of HCC patients. Moreover, the significance of clinical characters in the clustering of HCC subtypes were evaluated *via* Pearson Chi-square test, and significant differences of the phase of tumor metastasis (N classification, *P*<0.05; T classification, *P*<0.001), age (*P*<0.01), tumor grade (*P*<0.001) and gender (*P*<0.001) were found between two clusters (**Figure 2B**). We found that the patients with high clinicopathological grade were significantly concentrated in cluster 2. The OS rate was also assessed between two clusters, and cluster 1 exhibited the superior survival status to cluster 2 with significant difference (**Figure 2C**). Thus, HCC patients could be well categorized into two clusters associated with distinct survival rates based on expression levels of the PRGs.

### Construction of the DEGs prognostic model

**Figure 3:**
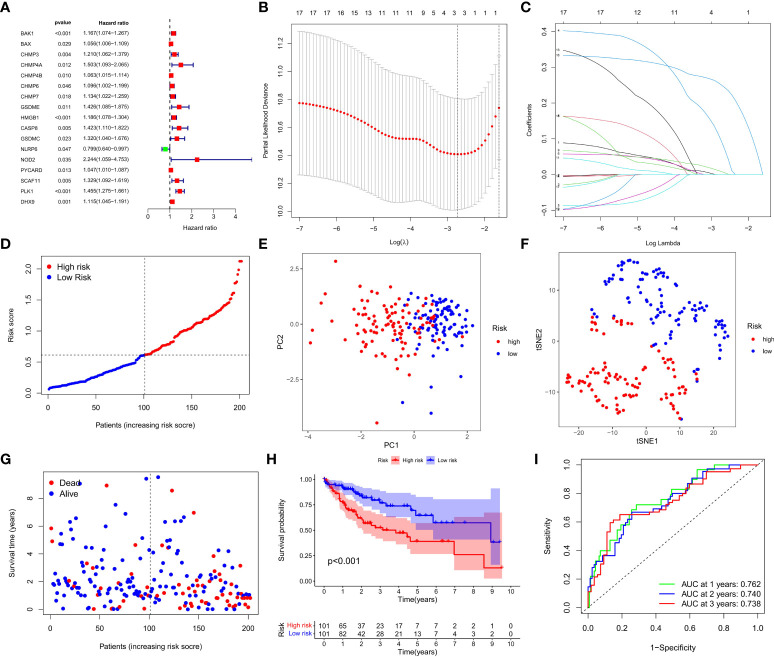
Construction of the DEGs prognostic model. **(A)** Univariate Cox regression analysis was used to assess DEGs of 202 HCC samples in the training cohort, and 17 genes were screened out upon P<0.05. **(B)** Cross validation of the 17 genes was performed for tuning the parameter selection in the LASSO regression. **(C)** 17 genes were assessed in LASSO Cox regression for the construction of a prognostic model. **(D)** Patients in training cohort were arrayed based on the risk score. **(E)** PCA for survival status was plotted based on the risk score. **(F)** T-SNE for survival status was plotted based on the risk score. **(G)** The survival status of patients was shown in the high- and low-risk population. **(H)** Kaplan-Meier survival curves of patients were displayed in high- and low-risk groups. **(I)** ROC curve was used to evaluate the predictive efficacy of the prognostic model.

A total of 404 HCC samples that had intact survival time records in the corresponding patients from the TCGA database were equally and randomly split into the training and test cohorts. We compared the two cohorts by Chi-square test and one-way ANOVA analysis and found no significant difference between them in the clinical characters including age, gender, the phase of tumor metastasis, clinical stage, degree of tumor differentiation. Univariate Cox regression analysis was conducted to evaluate the prognostic value of each DEG in the training cohort, and 17 survival-related DEGs matching the minimum criteria of *P*<0.05 were primarily screened (**Figure 3A**). Then, the least absolute shrinkage and selection operator (LASSO) Cox regression analysis was performed to regulate the parameter selection for the avoidance of DEGs overfitting through cross validation, and construct a 3-gene signature (*CHMP4A*, *HMGB1*, and *PLK1*) prognostic model based on the optimum λ value (**Figures 3B, C**). The risk score in the prognostic model was calculated with the established formula: risk score = (0.0926×*CHMP4A* exp.) + (0.0082×*HMGB1* exp.) + (0.2503×*PLK1* exp.). Based on the median risk score, 202 HCC patients in the training cohort were equally divided into high- and low-risk groups (**Figure 3D**). Principal component analysis (PCA) and t-distributed stochastic neighbor embedding (t-SNE) analysis were performed to confirm that patients with different risks could be well classified into two clusters corresponding to low- and high-risk groups respectively (**Figures 3E, F**). Patients in the low-risk groups showed fewer deaths and longer survival time than those in the high-risk group (**Figure 3G**). Kaplan-Meier analysis further evidenced significant difference of OS time between two groups, demonstrating the higher living probability in low-risk group than that in high-risk group (*P*<0.001, **Figure 3H**). Furthermore, time-dependent receiver operator characteristic (ROC) analysis was employed to evaluate the accuracy and precision of the risk model. The area under the ROC curve (AUC) was calculated as 0.762 in 1 year, 0.740 in 2 years, and 0.738 in 3 years (**Figure 3I**), indicating the reliability and feasibility of DEGs signature in the prognostic model. Taken together, these findings highlight that a three-PRG prognostic model comprising CHMP4A, HMGB1 and PLK1 was constructed in the TCGA training cohort.

### Validation of the prognostic model in the TCGA test group and ICGC group

**Figure 4:**
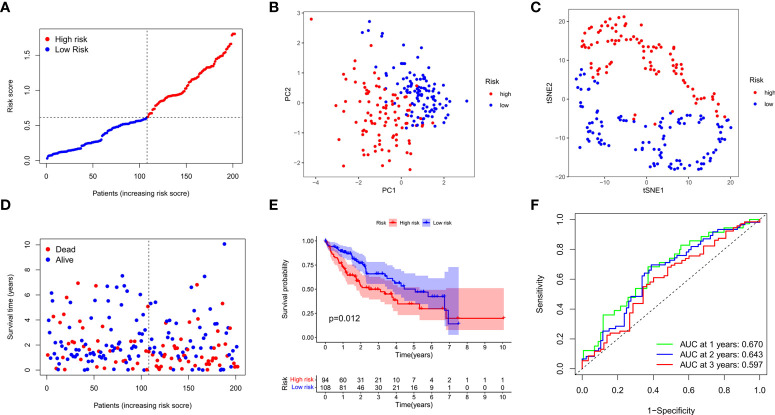
Validation of the prognostic model in the test cohort. **(A)** Distribution of patients in the test cohort was shown based on the median risk score in the training group. **(B)** PCA was plotted for survival status of HCC patients in the test cohort. **(C)** T-SNE analysis was conducted for survival status of HCC patients in the test cohort. **(D)** The survival status for each patient was displayed in the high- and low-risk groups. **(E)** Kaplan-Meier survival curves between low- and high-risk groups were shown in the test cohort. **(F)** ROC curves were assessed for survival status corresponding to 1, 2, and 3 years respectively.

**Figure 5:**
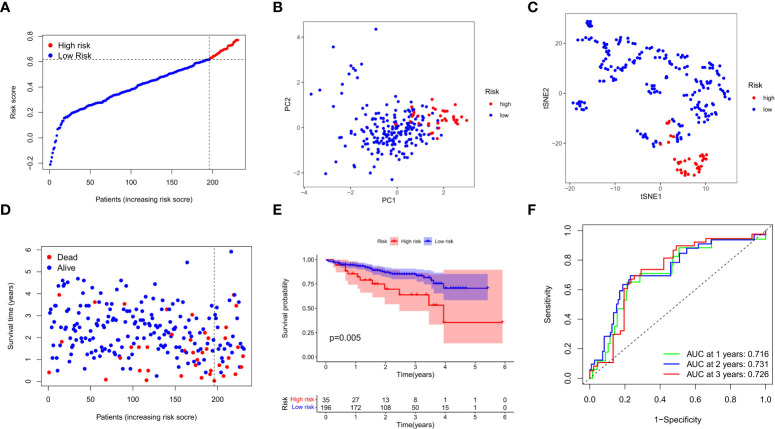
External validation of the prognostic model in the ICGC cohort. **(A)** Distribution of the risk scores in the ICGC cohort. **(B)** The PCA analysis for survival status of HCC patients in the ICGC cohort. **(C)** T-SNE analysis was conducted for survival status of HCC patients in the ICGC cohort. **(D)** The survival status for each patient was displayed in the high- and low-risk groups. **(E)** Kaplan-Meier survival curves between low- and high-risk groups were shown. **(F)** ROC curves were assessed for survival status corresponding to 1, 2, and 3 years respectively.

We next verified the efficiency of the prognostic model in the TCGA test cohort that included 202 HCC cases and ICGC external validation cohort. Gene expression data were first standardized by the “Scale” function, and risk scores of these patients were calculated with the established formula. According to the median risk score calculated in the training cohort, these cases were separated into two groups, with 108 patients in low-risk group and 94 in high-risk group (**Figure 4A**). PCA and t-SNE analysis exhibited the well separation of these patients into two subgroups significantly associated with survival rate based on the DEGs signature in the prognostic model (**Figures 4B, C**). Low-risk patients displayed lower death rate and higher survival probability than high-risk ones (**Figure 4D**). Kaplan-Meier analysis demonstrated that the OS time of high-risk patients was significantly shorter than that of low-risk ones (*P*=0.012, **Figure 4E**). ROC curve analysis also confirmed the good predictive efficiency of the prognostic model with the manifestation of AUC as 0.670 for 1 year, 0.643 for 2 years, and 0.597 for 3 years respectively (**Figure 4F**). The ICGC external validation cohort was spilt into the high- and low-risk groups based on the risk score (**Figure 5A**). The PCA and t-SNE analysis indicated that the risk genes were well able to separate the two risk groups (**Figures 5B, C**). High-risk patients displayed high death rate and lower survival probability than low-risk ones (**Figure 5D**). The Kaplan-Meier analysis suggested that the OS time of the low-risk group was higher (*P*=0.005, **Figure 5E**), and the model was reliable of AUC as 0.716 for 1 year, 0.731 for 2 years, and 0.726 for 3 years (**Figure 5F**). Overall, these findings suggest that prognostic model could be validated well in the TCGA Test Group and ICGC Group.

### Independent predictive value of the prognostic model

**Figure 6:**
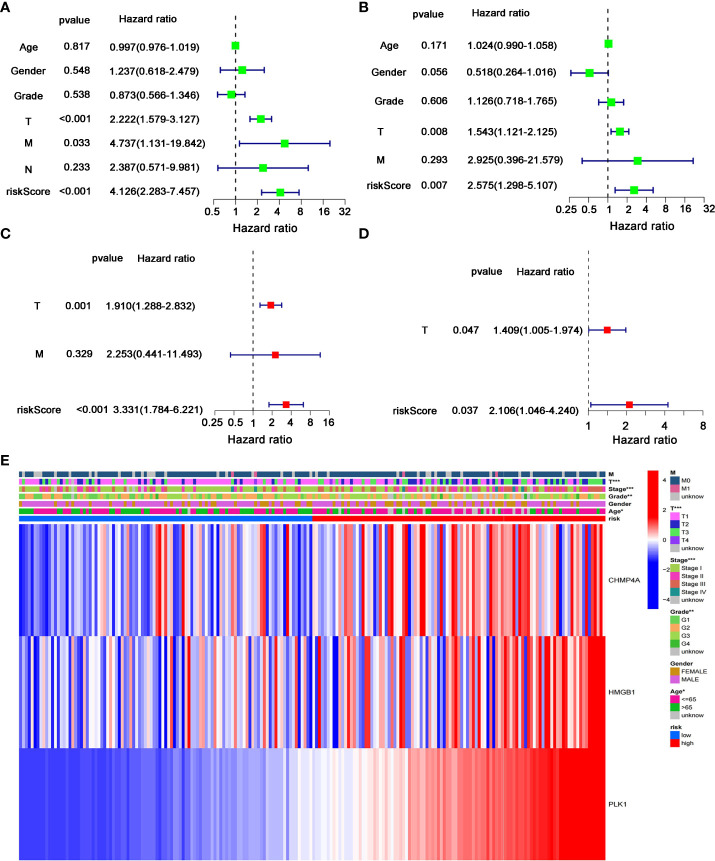
Independent predictive value of the prognostic model. **(A, B)** Univariate and **(C, D)** multivariate Cox regression analysis was separately used to assess the significance and hazard ratio value of risk score and clinical characters of HCC patients in the **(A, C)** training cohort and **(B–D)** test cohort. **(E)** Heatmap was presented to display the connections between clinicopathologic features and the risk groups. *P < 0.05; **P < 0.01; ***P < 0.001.

We next performed the univariate and multivariate Cox regression analyses to investigate whether risk score originated from the DEGs prognostic model could act as the independent factor predicting the survival status of HCC patient. The univariate Cox regression analysis showed that high risk score was an independent prognostic factor for poor survival of patients in the training cohort (*P*<0.001, HR=4.126, 95% CI: 2.283-7.457, **Figure 6A**) and test cohort (*P*=0.007, HR=2.575, 95% CI: 1.298-5.107, **Figure 6B**). The multivariate Cox regression analysis also indicated that, after normalized adjustment of variables, risk score was a prognostic factor independent on other clinical factors for HCC patients in the training cohort (*P*<0.001, HR=3.331, 95% CI: 1.784-6.221, **Figure 6C**) and test cohort (*P*=0.037, HR=2.106, 95% CI: 1.046-4.240, **Figure 6D**). Moreover, the univariate and multivariate Cox regression analyses revealed the significant correlation of T staging in tumor metastasis to the prognostic survival of HCC patients in both cohorts (**Figures 6A-D**). In addition, the heatmap that integrated the expression of DEGs signature and clinical characters of HCC patients revealed the significant difference of clinical stage, age, T classification and tumor grade between the low- and high-risk groups (*P*<0.05, **Figure 6E**). Collectively, these data supported the notion that the risk score of this model was confirmed as an independent prognostic factor to predict OS of HCC patients.

### Analyses of gene function and signaling based on the prognostic model

**Figure 7:**
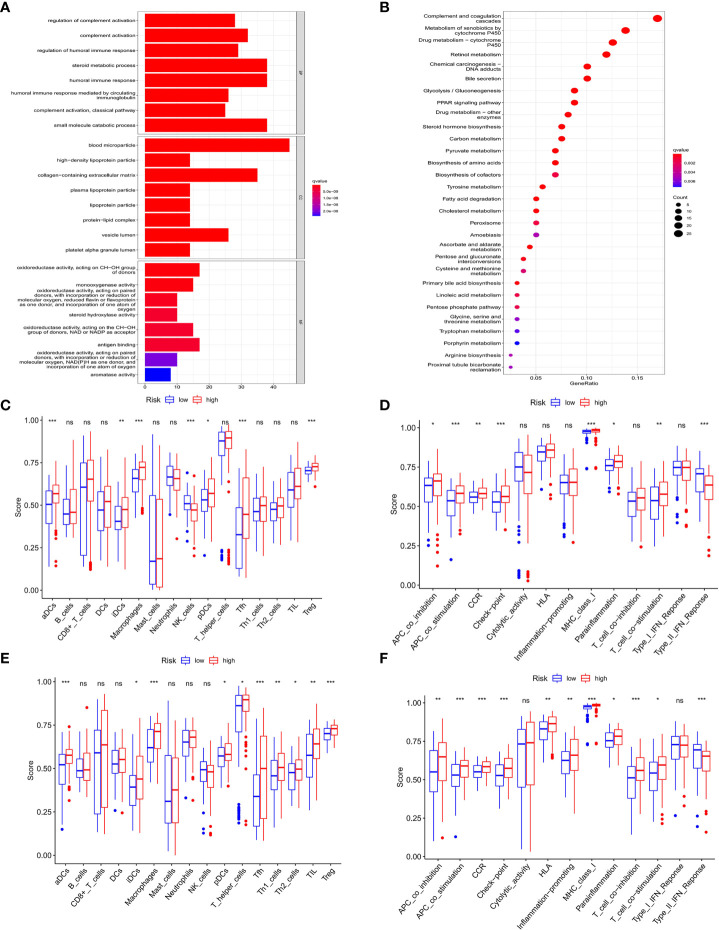
Functional analysis of DEGs in HCC and association of immune constitution with risk score in patients. **(A)** Barplot graph displayed GO enrichment, with the longer bar represented as the more enriched genes, and the increasing depth of red as the more obvious difference. **(B)** Bubble graph displayed KEGG analysis, with the bigger bubble represented as the more enriched genes, the increasing depth of red as the more obvious difference, and q-value as the adjusted p-value. **(C-F)** Comparison of the enrichment of diverse immune cells **(C–E)** and immune-related pathways **(D–F)** between low (blue box) and high risk (red box) group in the training cohort **(C, D)** and test cohort **(E, F)**. ns, no significance; *P < 0.05; **P < 0.01; ***P < 0.001.

We further investigated the difference of gene function and signaling between the two groups clustered by the PRGs prognostic model. Using the “limma” R package, a total of 2858 DEGs were extracted and identified between the low- and high-risk groups in the training cohort upon the criteria of FDR<0.05 and |log_2_FC|>2. Gene ontology (GO) enrichment analysis and Kyoto Encyclopedia of Genes and Genomes (KEGG) pathway analysis were performed to assess the assortment and functional characters of these DEGs. The results of GO analysis indicated that the DEGs were mainly involved in the humoral and complement immune response, glucose and lipid metabolic signaling, and oxidoreductive regulatory process (**Figure 7A**). Moreover, KEGG pathway analysis demonstrated the enrichment of DEGs in the complement and coagulation cascade, and multiple metabolism processes (**Figure 7B**).

### Comparison of the immune activity between the high- and low-risk groups

To explore the connection of immune activity with survival status in the two groups divided by risk scores in the prognostic model, ssGSEA was employed to compare the immune cell infiltrations and immune-related signaling pathways between low- and high-risk groups in both cohorts. In the training cohort, the significant difference occurred in the subsets of activated dendritic cells (aDCs), induced dendritic cells (iDCs), macrophages, natural killers (NK) cells, plasmacytoid dendritic cells (pDCs), follicular helper T cells (Tfhs), and regulatory T (Treg) cells between two groups, while other immune cells showed no remarkable alteration (**Figure 7C**). Notably, the fact that the numbers of macrophages and Tregs in the high-risk group were more than those in the low-risk group manifested the downregulation of immune activity in high-risk patients. In addition, the scores of APC co-inhibition, CCR, check point, parainflammation and T cell co-stimulation were higher in the high-risk group than those in the low-risk group, while type II IFN response signaling showed the converse result (**Figure 7D**). Similar results were also shown in the test cohort that aDCs, iDCs, macrophages, pDCs, Tfh, Th1, Th2, TIL and Treg cells exhibited the increased activity in high-risk patients than those in low-risk ones (**Figure 7E**). For the change of immune-related signaling, the condition in the test cohort was consistent with those in the training cohort (**Figure 7F**).

### Validation of the differential expression of the three prognostic genes

**Figure 8:**
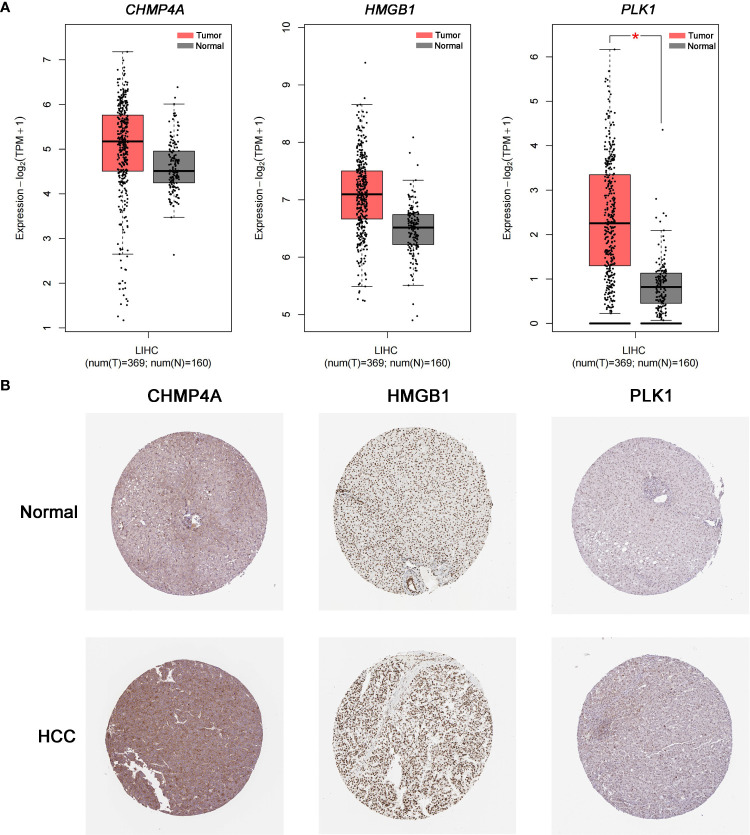
Validation of the differential expression of the three prognostic genes in clinical samples. **(A)** The mRNA levels of *CHMP4A*, *HMGB1* and *PLK1* were detected in normal tissues and HCC tissues from the GEPIA *P < 0.05. **(B)** Immunohistochemistry of the CHMP4A, HMGB1 and PLK1 was determined in HCC tissues and normal tissues from the HPA database.

**Figure 9:**
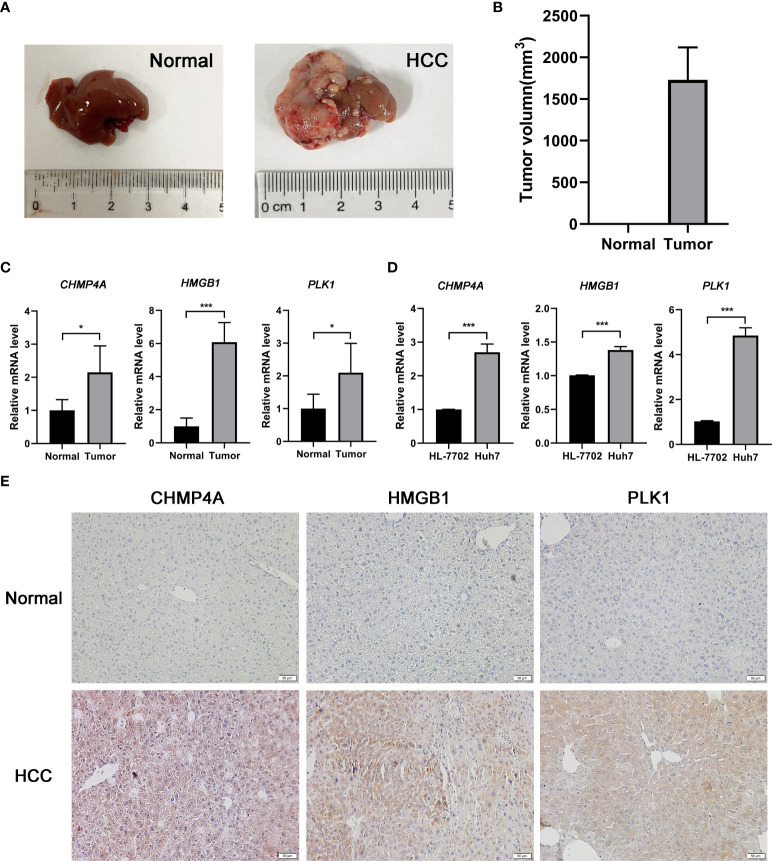
Validation of the differential expression of the three prognostic genes *in vivo* and *in vitro*. **(A)** Typical diagrams of normal and tumor liver tissue from mice were presented. **(B)** Tumor volumes between normal and HCC models from mice were measured. (n = 5, per group) **(C)** The mRNA levels of *CHMP4A*, *HMGB1* and *PLK1* were detected in the normal and HCC livers of mice. (n = 5, per group) **(D)** The mRNA levels of *CHMP4A*, *HMGB1* and *PLK1* were assessed between HL-7702 and Huh7 cells. (n = 3) *P < 0.05; ***P < 0.001. **(E)** The expression of CHMP4A, HMGB1 and PLK1 was determined in HCC tissues and non-tumor tissues from human samples performed by immunohistochemistry.

The expression difference of the three genes between normal and HCC tissues were verified by GEPIA (**Figure 8A**). As expected, the mRNA levels of *CHMP4A*, *HMGB1* and *PLK1* were significantly increased in HCC tissues rather than normal ones. Moreover, the protein levels of these three genes were detected using the human protein atlas (HPA) database, and the result showed the significant higher expression of CHMP4A, HMGB1 and PLK1 in the tumor tissues than in the normal ones (**Figure 8B**). We further corroborated the significant increase of these genes expression in HCC cells using *in vivo* and *in vitro* models. Tumor formation was obviously observed in orthotopic HCC model, and the dramatic increase of tumor volume was recorded (**Figures 9A, B**). In parallel, RT-qPCR detection demonstrated the transcription levels of *CHMP4A, HMGB1* and *PLK1* were increased in tumor tissues compared to normal ones (**Figure 9C**). Additionally, we also compared the mRNA levels of these three genes in the human HL-7702 hepatocytes with those in human Huh7 HCC cells, and the results manifested the superior increase of these genes expression in Huh7 cells in contrast to HL-7702 hepatocytes (**Figure 9D**). Moreover, the expression levels of these three genes were detected using immunohistochemistry assay, and the result showed the higher expression of CHMP4A, HMGB1 and PLK1 in the tumor tissues than in the normal ones (**Figure 9E**). Collectively, these data supported the notion that CHMP4A, HMGB1 and PLK1 are closely association with the prognosis of HCC.

## Discussion

HCC is one of the leading contributors to the cancer burden worldwide accounting for 90% of liver cancer cases (29). The improvement of therapeutic avenues such as surgical resection, chemotherapy, and radiotherapy posed limited benefit for the decrease of HCC mortality (30). At present, α-fetoprotein (AFP) measurement is the conventional estimated standard for the diagnosis and prognosis of HCC patients; however, the testing effect is not sensitive and specific enough due to the interference of HCC-unrelated factors and tumor heterogeneity (31). Therefore, the current situation necessitates the finding of efficient diagnostic and prognostic markers to help HCC patients in improving their clinical outcome. Since it was defined to be a novel pro-inflammatory programmed cell death in 2001, pyroptosis has gained increasing interest in the tumor research (13). However, little has been elucidated on the prognosis and relevant mechanism of PRGs in HCC.

In this study, we performed the RNA expression screening analysis using a TCGA dataset and discovered a total of 46 differentially expressed PRGs between normal tissues and HCC, among which only 4 genes were significantly downregulated in HCC while other 42 genes were upregulated. Based on these DEGs, the consensus clustering analysis could separate HCC patients into two subtypes, which exhibited significant differential survival rate, proving prominent value for clinical evaluation and assortment. Subsequently, univariate and LASSO Cox regression analyses were carried out to establish a PRGs-based independent risk model to predict the prognosis of HCC patients, which was well validated in the test cohort and ICGC external validation cohort. This model yielded significant survival difference by assigning patients into different risk groups. The results of ROC curve analysis further consolidated the predictive feasibility of this prognostic model. Pyroptosis is considered to be associated with inflammation and tumor immunity (32), thereby we explored the correlation of the risk model and immune activity in HCC patients. The significant accumulation of macrophages and Treg cells, and the significant decrease of type II IFN response were observed in high-risk patients in the both training and test cohort, indicating the poor prognosis was possibly due to the immunocompromised effect. Considering that the independent prognostic model was constructed using *CHMP4A*, *HMGB1*, and *PLK1*, we then validated their differential expressions in the normal and HCC tissues. Both mRNA and protein of these genes displayed higher level in HCC tissues than in normal ones in the clinical samples, as evidenced by GEPIA and HPA. The similar tendency of gene expression was also validated *in vivo* mouse HCC model, *in vitro* human HCC cells and human samples.

The prognostic model included three PRGs: *CHMP4A*, *HMGB1*, and *PLK1*. CHMP4A is the member of the chromatin-modifying protein/charged multivesicular body protein (CHMP) family, and an essential component of the mammalian endosomal sorting complex required for transport III (ESCRT-III), which is involved in the degradation of membrane protein and the resistance to cell death (33). It has been reported that the level of CHMP4A was markedly increased in men with high GS prostate cancer, and in patients with advanced high-grade serious ovarian cancer (HGSOC), and thereby it could act as a potential biomarker for cancers (34, 35). In HCC, CHMP4A was found to be highly expressed, and involved in pyroptosis (36). Some studies demonstrated that CHMP4A promotes mitotic cytokinetic, midbody abscission and multivesicular body organization, which play the functional role in involving viral infection partly related to the significance of ESCRT-III in the HCC progression (33). We revealed that the expression level of CHMP4A was associated with poor prognosis, which may be due to its regulation of pyroptosis. Accumulating evidence has shown that hypoxia is a common phenomenon in the growth of solid tumor, and CHMP4A acts as the modulator to increase the expression level of hypoxia-inducible factor-1 α protein (HIF-1α), promoting the tumor progression in a hypoxia-associated pro-tumor mechanism (37). HMGB1 is a non-histone chromatin-associated protein widely distributed in eukaryotic cells and transferred to extracellular space as a danger alarm. It was overexpressed in various types of cancers, and played a key role in the regulation of inflammation and cancer progression (38). It was reported that GSDME-mediated pyroptosis triggered the release of HMGB1, leading to the development of colitis-associated colorectal cancer (CAC) *via* the activation of ERK1/2 signaling pathway (39). Moreover, HMGB1 functioned as the executor of pyroptotic cell death to participate in the regulation of tumor immune microenvironment *via* the inhibition of mutant BRAF and MEK (40). High expression of HMGB1 was found in lung cancer tissues, and its release upon pyroptotic cell destruction could be used as a prognostic marker of survival (41). In breast cancer, HMGB1 translocation was demonstrated to be associated with pyroptosis (42). In HCC, HMGB1 was proved to interact with mitochondrial DNA to promote the activation of toll-like receptor (TLR)-9 signaling in hypoxia condition for the stimulation of tumor development (43). Studies have shown that HMGB1 can form an RNA-RNA crosstalk network with RICTOR, promote the progression of liver cancer by promoting glutamine metabolism, and enhance the activity of PD-L1 exosomes to reduce tumor immunotherapy (44). In addition, studies also showed that HMGB1 release serves as the marker of immunogenic cell death, and induces the cytotoxic T immune response to cancer by activating antigen-presenting dendritic cells (45). Furthermore, HMGB1 is closely related to cancer immunity microenvironment, and exosome-derived HMGB1 can promote the expansion of TIM-1^+^ Regulatory B cells through TIL2/4 and MAPK signaling pathways, resulting in immune escape in HCC (46). Our results showed that HMGB1 was increased in HCC tissues and correlated with poor outcomes, further confirming the potential as a prognostic biomarker of HCC patients. PLK1 is a type of serine-threonine kinase that plays a key role in the regulation of cell cycle and the maintenance of genome stability (47). Increasing evidence demonstrated that PLK1 was overexpressed in various malignant tumors, and its upregulation was associated with the poor prognosis of cancer patients (48, 49). In mechanism, PLK1 could bind to STK39 to activate ERK signaling, which promote HCC progression (50). In addition, PLK1 could be highly co-expressed with BIRC5 in p53-mutated HCC, and thereby co-inhibition of PLK1 and BIRC5 synergistically compromised the viability of p53-mutated HCC cells *in vitro* and *in vivo* (51). FBXO45 induced liver tumorigenesis *via* PLK1 upregulation, indicating PLK1 is a promising target for HCC therapy (52). Moreover, PLK1 was also found to involve tumor immunity *via* triggering the TGF-β signaling pathway, and induce immune escape in favor of lung cancer metastasis by promoting the expression of PD-L1 (53). Under hypoxic conditions, PD-L1 is translocated to the nucleus and together with p-Stat3 regulates the transcription of GSDMC, resulting in the conversion of apoptosis to pyroptosis after activation of caspase-8 by TNFα (54). Additionally, studies have shown that PLK1 can promote the progression and metastasis of clear cell renal cell carcinoma by upregulating hypoxia-inducible factor 2, which transcriptionally targeting the hypoxia-responsive element of the PLK1 promoter (55). Here, we revealed that the level of PLK1 was also increased in HCC and significantly associated with the poor prognosis of HCC patients, confirming the significance of PLK1 as a risk factor in the evaluation of survival of HCC patients.

In this study, we identified several prognostic PRGs and built a novel pyroptosis-related prognostic model for HCC patients. Nevertheless, our research still has some limitations. Firstly, our research results were based on retrospective analysis, which should be further verified in prospective studies. Second, single-gene diagnosis is relatively difficult and inaccurate. Third, immune-related research has not been well validated in this model, which inspired us to explore the prognostic value of pyroptosis by using multiple DEGs.

## Conclusion

In summary, we built a novel independent risk model consisting of three PRGs, and validated the efficiency of this model in predicting the prognosis of HCC patients. Thus, our work shed important insight into the survival prediction of HCC, and provided several promising targets for HCC therapy.

## Data availability statement

The original contributions presented in the study are included in the article/Supplementary Material. Further inquiries can be directed to the corresponding authors.

## Ethics statement

This animal study was reviewed and approved by the animal experimentation ethics committee of Shanghai University of Traditional Chinese Medicine. The studies involving human participants were reviewed and approved by the Clinical Research Ethics Committee of Shanghai University of Traditional Chinese Medicine. The patients provided their written informed consent to participate in this study.

## Author contributions

YYL designed the experiments, analyzed the data and wrote the manuscript. YL acquired the data and performed the experiments. XZ and XD analyzed the data. HF, ZY and YG critically revised the article. All authors contributed to the article and approved the submitted version.

## Funding

This work was supported by National Natural Science Foundation of China (No. 82222074, 81874436, 82074154, 81774240), Three-year Action Plan for the Development of Chinese Medicine in Shanghai (No. ZY (2018-2020)-CCCX-2003-01), Shanghai Key Clinical Specialty Construction Project (No.shslczdzk01201), The Siming Scholar from Shanghai Shuguang Hospital (SGXZ-201904), Youth Tip-top Talent program in Shanghai, Xinglin Youth Scholar from Shanghai University of Traditional Chinese Medicine.

## Acknowledgments

We would like to acknowledge the TCGA and ICGC database, GEPAI and HPA database. We also thank all our authors listed in this manuscript.

## Conflict of interest

The authors declare that the research was conducted in the absence of any commercial or financial relationships that could be construed as a potential conflict of interest.

## Publisher’s note

All claims expressed in this article are solely those of the authors and do not necessarily represent those of their affiliated organizations, or those of the publisher, the editors and the reviewers. Any product that may be evaluated in this article, or claim that may be made by its manufacturer, is not guaranteed or endorsed by the publisher.
